# Anaesthetic management of oncological disease in pregnancy: a narrative review

**DOI:** 10.1111/anae.16489

**Published:** 2025-01-08

**Authors:** Ben Sharif, Melanie Nana, Rachel Kearns, Queenie Lo, Yavor Metodiev

**Affiliations:** ^1^ Department of Anaesthesia University Hospital of Wales Cardiff UK; ^2^ Department of Obstetric Medicine St Thomas' Hospital London UK; ^3^ Department of Anaesthesia Glasgow Royal Infirmary Glasgow UK; ^4^ School of Medicine University of Glasgow Glasgow UK; ^5^ Department of Anaesthesia and Perioperative Medicine The Royal Marsden Hospital NHS Foundation Trust London UK

**Keywords:** anaesthesia, cancer, oncological disease, pregnancy

## Abstract

**Introduction:**

Cancer complicates approximately 1 in 2000 pregnancies, with increasing incidence due to factors such as increased maternal age, obesity and advancements in antenatal testing. Anaesthetists play a crucial role in managing pregnant patients with cancer, both during delivery and in providing anaesthesia for oncological treatments. This review explores the challenges in anaesthetic management and specific considerations for common cancers encountered in pregnant patients.

**Methods:**

An electronic literature search was carried out using PubMed and Google Scholar to identify peer‐reviewed articles published in English from 1 January 1990 to 30 July 2024.

**Results:**

Two main areas were identified: anaesthetic management related to pregnancy and the peripartum period in patients with cancer; and oncological management during pregnancy. Current data suggest that pregnancy does not worsen cancer prognosis, but diagnosis and treatment are complicated by the overlap of cancer symptoms with physiological changes of pregnancy and concerns about the safety of diagnostic procedures and treatments. Ultrasound and magnetic resonance imaging are preferred imaging modalities, while careful use of ionising radiation is advised. Treatment during pregnancy, including surgery, chemotherapy and radiotherapy is possible, with specific timing and modality considerations to ensure maternal and fetal safety. Anaemia, poor nutrition and preterm birth are significant concerns in managing pregnant patients with cancer. For operative births, neuraxial techniques are preferred, though general anaesthesia may be required in complex cases. Comprehensive multidisciplinary support, including psychosocial care, is essential for optimal outcomes. Oncological surgery during pregnancy should preferably be scheduled for the second trimester, with consideration for fetal monitoring and steroids. Regional anaesthesia should be utilised if possible and uteroplacental perfusion maintained. Increased risks of thromboembolism should be addressed postoperatively, along with psychological support.

**Discussion:**

Effective and safe anaesthetic management of cancer in pregnancy requires a multidisciplinary approach to balance maternal and fetal safety, with a focus on careful planning and individualised care.

## Introduction

Around 1 in 2000 pregnancies are complicated by cancer, an incidence which is increasing over time [1]. Trends towards increasing maternal age, rising obesity rates and the implementation of non‐invasive antenatal testing are all likely to contribute to this increasing incidence [2, 3]. The role of the anaesthetist in the care of such patients is twofold; supporting safe intrapartum care and delivery; and providing anaesthesia for oncological management. This narrative review focuses on the impact of cancer during pregnancy, provides insight into the challenges and management for these patients and considers the specifics of some common cancers encountered.

## Methods

An electronic literature search was carried out using PubMed and Google Scholar to identify peer‐reviewed articles published in English from 1 January 1990 to 30 July 2024. Search terms included ‘pregnancy’; ‘oncology’; ‘neoplastic’; and ‘anaesthesia’. The articles selected for inclusion were those that, in the opinion of the authors, were of the greatest relevance to current clinical care.

## Results

We identified two broad areas of clinical interest concerning the pregnant patient with cancer: the anaesthetic management related to pregnancy; and the peripartum period and the oncological management during pregnancy.

### Incidence and treatment of cancer in pregnancy

In general, the types of cancers seen in pregnancy reflect those seen in women of child‐bearing age, with breast cancer, cervical cancer, ovarian cancer, lymphoma and melanoma diagnosed most frequently [4]. Diagnosis may be made at any stage of pregnancy but is most common in the second trimester [4]. The available data suggest that in most types of cancer, pregnancy itself does not worsen cancer prognosis [1]. However, concerns regarding the safety of the mother and fetus during investigations and treatment put women at risk of delayed diagnosis, later presentation and inappropriate treatment, which can culminate in poorer outcomes.

The diagnosis of cancer during pregnancy can be challenging as symptoms of malignancy often mimic common physiological symptoms of pregnancy [5]. The number of new cancer diagnoses during pregnancy is less than expected in population‐based numbers, with a Swedish registry‐based study of > 4.5 million births finding an ‘observed to expected case’ ratio of 0.46 (95%CI 0.43–0.49), and a rebound increase in diagnoses has been observed in the postpartum period [6]. In 2021, the Confidential Enquiry into Maternal Deaths in the UK (MBRRACE) reported that 59/80 (74%) women with cancer in pregnancy could have had improvements to their care with key themes identified relating to recognition of concerning symptoms; avoiding normalisation bias; ensuring symptoms are investigated; and the appropriate staging of new cancers [7].

Ionising radiation for diagnostic imaging should be avoided if alternative techniques with equal accuracy are available [5]. If no alternatives exist, then the maximum cumulative fetal dose of 100 mGy is recommended to avoid adverse fetal outcomes. Most radiographic or computed tomography (CT) imaging fall well below this threshold, except for pelvic CT (Table 1) [8]. Abdominal shielding has no benefit in reducing fetal exposure from non‐pelvic imaging but may offer maternal reassurance [9]. Ultrasound and magnetic resonance imaging (MRI) are the only imaging modalities considered safe in all trimesters due to the absence of ionising radiation, with whole‐body diffusion‐weighted MRI feasible for detection of primary lesions as well as nodal and distal metastases [10].

**Table 1:** Fetal radiation exposure for radiography and computed tomography for each body region [8].

Imaging technique	Body region	Fetal radiation exposure (mGy)
Radiography	Chest	0.001–0.01
	Mammography	0.001–0.01
	Abdomen	0.1–1.0
	Pelvis	0.1–1.0
Computed tomography	Head	0.001–0.01
	Chest	0.1–1.0
	Pulmonary angiogram	0.01–0.1
	Abdomen (routine)	1–10
	Pelvis	10–50

Treatment of cancer during pregnancy is possible. It is preferable to perform surgery in the second trimester but should not be avoided at any gestation where it will have impact on the patient's outcome. Chemotherapy drugs are teratogenic in the first trimester but can be used with confidence after this period [11, 12]. In a cohort study of 1170 women over a 20‐year period, 779 (67%) received treatment during pregnancy with rates of chemotherapy seen to increase over the study period [4]. This was associated with more live births and fewer preterm births than at the beginning of the study period, though with increases in small‐for‐gestational age births and admissions to neonatal intensive care units [4]. Long‐term follow‐up studies of children exposed to chemotherapy have been reassuring [13]. Radiotherapy is not absolutely contraindicated in pregnancy but should only be performed with the support of an expert. It can be used in some cases, typically in the first or second trimester where the beam is directed away from the fetus with appropriate fetal shielding [12].

Increasingly reassuring data regarding treatment with surgery, chemotherapy and, in some cases, radiotherapy weighed against the significant short‐ and long‐term risks of preterm birth, means that in many cases treatment during pregnancy will ensure the best outcome for both mother and child [14, 15]. Endocrine therapy, targeted therapies and immunotherapy should be reserved for the postpartum period as there is currently not enough evidence to confirm their safety. Cancer during pregnancy requires multidisciplinary teams to deliver optimal and co‐ordinated care. This includes specialists in oncological diagnostics and management as well as clinicians who do not care routinely for patients with cancer [9]. Additionally, patients often require concurrent psychosocial support, and input may be needed from legal and ethical advisors. It is prudent that an obstetric anaesthetist is involved early in the management and planning of birth for patients with concurrent cancer.

### Anaesthetic management of pregnancy in patients living with cancer

**Figure 1:**
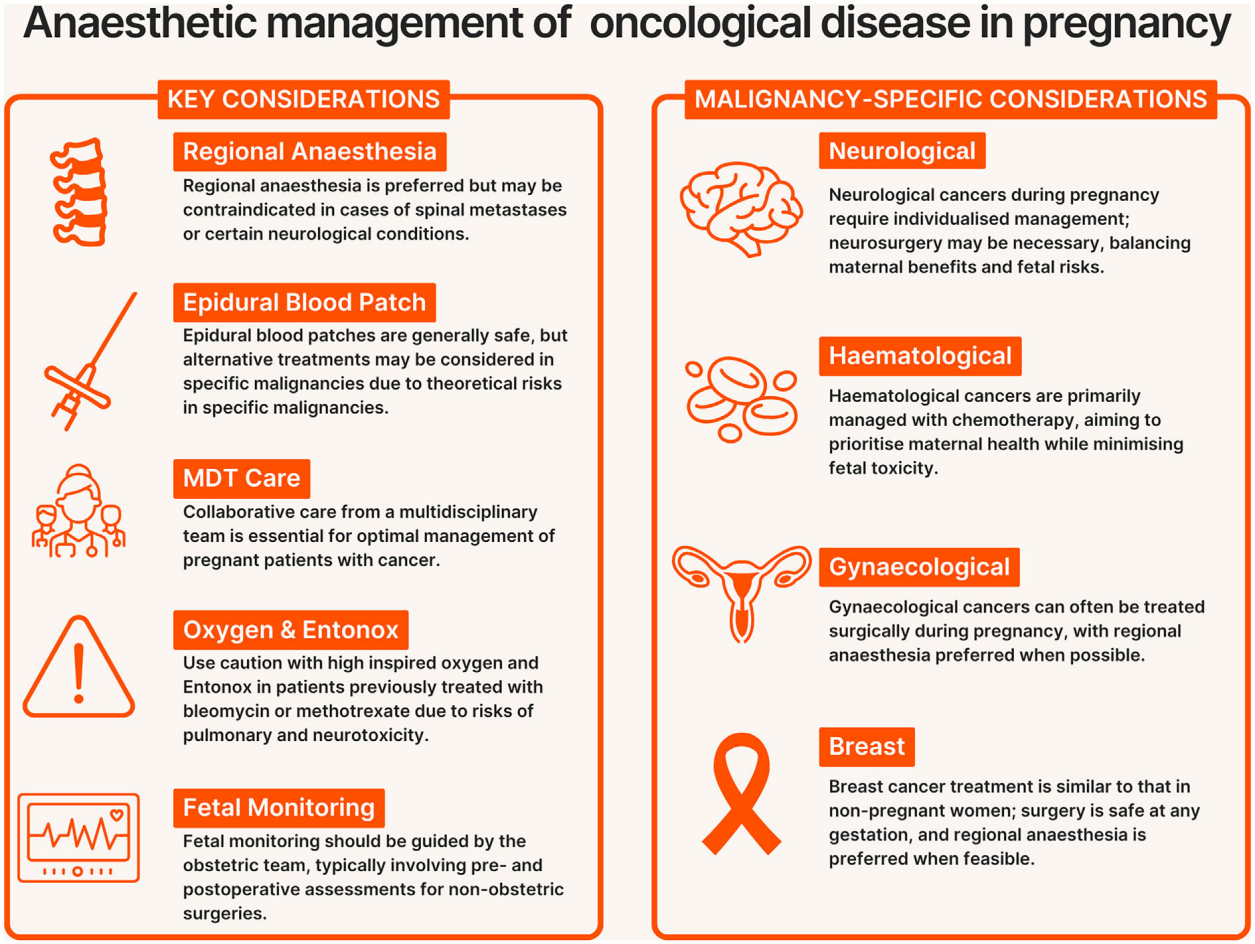
Infographic of anaesthetic considerations in the management of oncological disease in pregnancy.

Key elements are summarised in Figure 1. One‐third of patients with cancer have anaemia at diagnosis, and this is associated with impaired physical function and quality of life. Iron deficiency anaemia during pregnancy is common and can lead to worse fetal and maternal outcomes and an increased risk of postpartum haemorrhage [16]. Therefore, in pregnant patients with cancer, anaemia should be identified and treated. In the absence of response or intolerance to oral therapy [16], intravenous iron should be considered [17]. Poor nutritional state is a risk factor for postoperative complications and can be caused by the increased metabolic demand of tumours, physical obstruction within the gastrointestinal tract or the adverse effects of chemotherapeutic drugs [18]. Nutritional assessments should be completed antenatally and metabolic abnormalities treated to reduce the risks associated with maternal undernutrition, such as low‐birth weight and pre‐term birth [19].

Premature birth is associated with higher rates of neonatal morbidity and mortality [20] and antenatal exposure to most maternal cancers, with or without treatment, has not been found to impair the development of children in early childhood [21]. As a result, delivery should be planned for > 37 weeks gestation, unless there is maternal clinical deterioration or lack of response to oncological treatment [10]. Birth within 3 weeks of chemotherapy administration should be avoided. This allows placental metabolism of chemotherapy drugs and resolution of myelosuppression [10].

Vaginal birth is preferred due to reduced neonatal and maternal complications; however, a caesarean birth may be indicated in the presence of cervical or vulval cancers to avoid potential seeding of cancer cells into episiotomy sites. Large cervical cancers may warrant a classical uterine incision [22] and caesarean surgery may be utilised as an opportunity for concurrent hysterectomy or pelvic lymphadenectomy [23]. In the absence of signs of raised intracranial pressure, caesarean or vaginal birth with a neuraxial technique should be considered in patients with intracranial tumours to avoid elevations in intracranial pressure associated with uterine contractions and pushing in the second stage of labour [24]. Similar recommendations are reasonable for patients with bony metastases to avoid long bone fractures in labour [25].

Vertebral canal metastases, which can be asymptomatic or undiagnosed in the antenatal period, pose additional risks with neuraxial procedures, such as epidural catheters or single‐shot spinal anaesthetics. The evidence consists mainly of sporadic case reports ranging from uneventful procedures [26, 27] to devastating neurological injuries, such as acute paraplegia [28, 29, 30, 31, 32]. The presence of an obstructing mass can reduce the compliance of the epidural space and even a small volume of local anaesthetic can lead to a critical increase in the pressure within the epidural space [28]. In the presence of a continuous epidural infusion, stopping the infusion may lead to reversal of the paralysis [29].

Detection and treatment of cancer‐ or treatment‐associated thrombocytopenia is essential before delivery. Guidance on performance of neuraxial techniques is largely based on expert consensus, but a platelet count > 75 x 10^9^.l^‐1^ has been proposed as being adequate [33]. Both cancer and pregnancy increase the risk of venous thromboembolism. Prophylactic anticoagulation should be considered in these patients, typically with low molecular weight heparin, and timing of neuraxial techniques should be informed by existing guidelines [33]. Haemostatic abnormalities associated with cancer are diverse with many coagulation mechanisms potentially affected. Thromboembolic events are more common in patients with solid tumours, while patients with acute leukaemia are more prone to haemorrhagic events [34]. Quantitative and qualitative platelet abnormalities are often present, and these should be considered carefully when choosing a neuraxial technique [33].

Patients with cancer, particularly those who are immunosuppressed, are at greater risk of complications, such as transfusion‐associated graft versus host disease, due to blood product transfusion [35]. The use of leucocyte‐reduced and irradiated blood products should be considered [18]. In anticipation of expected blood loss, intra‐operative cell salvage is utilised occasionally in obstetric surgery. Clinicians should be reassured that cell salvage in combination with leucocyte depletion filters is considered safe and will not risk further spread of malignant cells [18].

Remifentanil patient‐controlled analgesia is suitable in clinical situations where a neuraxial analgesic technique is contraindicated. Its use is well established and high maternal satisfaction has been reported [36]. Remifentanil patient‐controlled analgesia use may lead to respiratory depression and apnoea which may not be tolerated by patients with intrathoracic masses or pathology [36]. Supplemental oxygen is often needed, but high fractions of inspired oxygen should be avoided in patients with a history of treatment with bleomycin due to risk of pulmonary toxicity [18]. Entonox is a mixture of 50% nitrous oxide and 50% oxygen which is used commonly for labour analgesia. There may be concerns over potentiating methotrexate neurotoxicity by the concurrent use of nitrous oxide, albeit most evidence coming from paediatric populations [37]. In addition, a history of bleomycin treatment should also raise concerns over using Entonox for a prolonged period due to its oxygen content.

In most cases, single‐shot spinal or combined spinal‐epidural anaesthesia are the preferred and routine techniques for operative births. However, neuraxial anaesthesia can be challenging due to the presence of vertebral pathology or coagulopathy. Additionally, total failure of spinal anaesthesia has been reported in patients who have received intrathecal chemotherapy, although a direct mechanism for this has not been identified [38]. Anaesthetists should be aware of the implications of intrathecal chemotherapy, especially if they are likely to perform subsequent neuraxial techniques [39].

General anaesthesia can be complicated in the pregnant patient living with cancer. Intrathoracic, mediastinal or airway masses or limitations in neck mobility due to cancer or radiotherapy can lead to challenges in airway and respiratory management. These are even more pronounced during pregnancy due to the physiological changes associated with pregnancy, such as airway oedema, decreased functional residual capacity of the lungs and higher metabolic rates and oxygen consumption, leading to shorter safe apnoeic periods [26].

Cardiotoxicity associated with chemotherapy is common and may develop over several years [18]. Dilated cardiomyopathy can be exacerbated by the increased intravascular volume associated with pregnancy and lead to poor outcomes [40].

Anaesthetic management of patients living with cancer in the postpartum period follows routine practice. The presence of active cancer or oncological treatment increases the risk of venous thromboembolism and postpartum pharmacological prophylaxis is usually indicated. Postoperative pain management can be challenging, especially in patients who are taking multiple long‐term opioids. A multidisciplinary approach should be embraced in the postpartum period and a thorough handover ensured between the obstetric and the oncology teams.

Postpartum patients living with cancer who develop postdural puncture headache should be treated according to local and national guidelines after evaluating any patient‐specific risks [41, 42]. A certain hesitancy exists regarding epidural blood patches due to concerns of seeding malignant cells; reassuringly, there are no documented cases of this occurring. Epidural blood patches are generally not contraindicated in the presence of malignancies. However, alternative treatments of postdural puncture headache may be considered in minimal residual disease in haematological malignancies, central nervous system tumours with increased intracranial pressure and poorly localised or metastatic tumours. These include non‐autologous blood for epidural injection, fibrin glue or sphenopalatine ganglion blocks [42].

### Anaesthetic management of oncological surgery during pregnancy

Where oncological surgery during pregnancy is considered necessary, meticulous planning is vital, recognising the relative rarity of this scenario and the lack of robust evidence on which to base recommendations. Surgery may take place at any point during pregnancy, though the preferred time window lies early in the second trimester to balance technical difficulties relating to the gravid uterus and potential risk of miscarriage in the first trimester [23].

Pre‐operatively, early involvement and consultation with senior members of the multidisciplinary team is essential to ensure that surgery is performed safely [9, 43]. Timing of surgery should consider the nature of the pathology, any neoadjuvant chemotherapy and gestation of pregnancy, ideally taking place several weeks after the cessation of chemotherapy and in the second trimester. Pre‐operative assessment should consider the details of chemotherapy or radiotherapy regimes and associated toxic effects such as dyspnoea, chest pain and fever. Routine testing should include blood tests to screen for anaemia or blood dyscrasias and identify any specific blood product or anti‐D requirements. Additional investigations may be required in women who have received cardiac‐ or pulmonary‐toxic chemotherapeutic drugs. Airway assessments should be performed with care if the woman has received radiotherapy to the head or neck. Prophylactic anticoagulation dose and timing should be ascertained before neuraxial procedures.

Before surgery, fetal monitoring should be performed under the direction of the obstetric team using fetal Doppler or cardiotocography according to gestation [43]. A strategy of performing only pre‐ and postoperative fetal monitoring is utilised commonly as intra‐operative monitoring may be difficult to interpret. Expectations of any actions resulting from non‐reassuring intra‐operative fetal monitoring should be discussed with the patient and the multidisciplinary team. Surgery during pregnancy is associated with an increased likelihood of preterm delivery [44] and the use of steroids for fetal lung maturation should be considered if delivery is thought to be likely [45]. Obstetric and neonatal services, along with essential equipment, should be on‐site and available at the time of surgery in the event there is an unanticipated need for fetal delivery (e.g. maternal cardiac arrest) [43, 46].

Intra‐operative management must consider the physiological changes of pregnancy. A left lateral tilt should be employed at ≥ 20 weeks gestation and the use of regional anaesthesia, where feasible, is recommended [45]. If general anaesthesia is necessary, a rapid sequence induction with pre‐oxygenation in the ramped position, videolaryngoscopy and a down‐sized tracheal tube are recommended. End‐tidal carbon dioxide should be maintained in the normal range for pregnancy (3.7–4.2 kPa) using a lung‐protective ventilation strategy. Blood pressure (and hence uteroplacental perfusion) should be maintained within 20% of baseline systolic levels [45]. In patients who have previously received bleomycin, the lowest concentration of inspired oxygen tolerated should be used to maintain oxygen saturation at between 88% and 92% to prevent bleomycin‐associated pulmonary fibrosis [18]. Evidence to support the use of TIVA over volatile anaesthetics to improve cancer outcomes is inconclusive and either technique may be used [47]. Intra‐operative compression stockings should be considered given the increased risk of venous thromboembolism in this patient group. Paracetamol, opioids and regional anaesthetic techniques may be used for analgesia, though non‐steroidal anti‐inflammatory drugs should be avoided after 32 weeks gestational age. Anti‐emetics should be administered routinely.

When considering a laparoscopic vs. open surgical approach, experience of the surgeon, gestational age, anticipated procedure time (< 90–120 min) and feasibility of maintaining low intra‐abdominal pressure (10–13 mmHg) should be considered. The patient should be monitored closely for hypercapnia, uterine perforation and increased intra‐abdominal pressure [48]. Postoperatively, the ongoing location of care will depend on the nature of surgery and the stage of pregnancy. Malignancy, associated therapies and pregnancy can trigger inflammation and a procoagulant response leading to venous thromboembolism. Therefore, thromboprophylaxis with low molecular weight heparin should be administered using a weight‐based regime for an extended period.

Pregnancy itself is a significant life event and a cancer diagnosis during pregnancy can be challenging for the patient and their family, with far‐reaching implications. Adequate ongoing psychological support must be offered and the support of charities, such as Mummy's Star in the UK, can be valuable. Women should be followed up in an anaesthetic clinic to discuss preparation for labour and birth, and consideration given to any further treatment required.

### Common cancers in pregnancy and anaesthetic considerations

In a multicentre cohort study of 1170 patients receiving treatment for cancer during pregnancy, the most common malignancies in pregnancy were reported as breast (39%) and cervical (13%), followed by lymphoma (10%) and ovarian (7%) [4].

Breast cancer is the most common malignancy in females and the leading cause of death in women aged 35–54 y [49]. Diagnosis during pregnancy or lactation occurs in approximately 1 in 3000 pregnant women with most cases diagnosed in the 6 months postpartum [49]. Although rare, the incidence of breast cancer during pregnancy is increasing, likely due to both a reduced age at diagnosis and an ageing obstetric population [50]. Literature suggests that pregnancy‐related breast cancer tends to present at a higher stage, most commonly with infiltrating ductal adenocarcinomas, which are often associated with more aggressive behaviour (high incidence of lymphovascular invasion and oestrogen receptor negativity) [11, 51, 52]. Delays in diagnosis and appropriate treatment due to pregnancy‐related physiological changes and concerns about radiation exposure and diagnostic tests can lead to poorer outcomes [53].

Treatment options should, where possible, reflect those of the non‐pregnant women. The main aim is to control the disease locally and prevent distant metastases, with surgery considered the safest treatment at any pregnancy stage. Breast‐conserving surgery or mastectomy can be performed based on tumour characteristics and breast size. Reconstruction is generally delayed avoiding prolonged anaesthesia time, although inserting a tissue expander during surgery may allow for later reconstruction. Radio‐isotope scintigraphy is not known to cause significant uterine radiation and is used for sentinel node assessment, but methylene blue dye is not recommended. Breast surgery aligns with the use of regional anaesthetic techniques for postoperative analgesia [49]. During the second trimester adjuvant chemotherapy may be administered, though radiotherapy is contraindicated until after delivery unless vital to conserving maternal life or organ function. If radiotherapy is essential, fetal shielding and early elective delivery should be considered. Hormonal treatments, such as tamoxifen, are not recommended. Pregnancy following breast cancer should be jointly supervised by an obstetrician, oncologist and breast surgeon, with particular attention to identifying any toxic effects of chemotherapy and planning for birth [49].

Cervical and ovarian cancers are the most common gynaecological cancers in pregnancy, with endometrial and vulval cancers occurring less frequently. Pregnancy does not adversely impact the prognosis of these malignancies and should be continued under multidisciplinary team care. Surgery is largely considered safe during pregnancy with regional anaesthetic techniques recommended, whenever possible.

Approximately two‐thirds of cervical cancer cases are diagnosed in the first and second trimesters [54]. Presenting symptoms, such as urinary frequency, pelvic or lower back pain and vaginal bleeding, can make it difficult to distinguish from pregnancy‐related symptoms, leading to delayed diagnosis. Suspected cervical cancer cases should be referred urgently to a gynaecologist and seen within 2 weeks [54]. Management of cervical cancer during pregnancy depends on tumour stage and size; nodal status; gestation; and histological subtype [55]. This can range from deferring surgery until after delivery in pre‐cancerous cases to immediate definitive treatment. Surgery during the second trimester is preferable due to a lower risk of miscarriage and improved surgical access. After radiological assessment and laparoscopic lymphadenectomy for tumour staging, surgery with or without neoadjuvant chemotherapy can be performed [54]. In cases of advanced disease or lymph node spread, the patient may choose not to preserve the pregnancy, based on local legislation and usually until 24 weeks of gestation. Psychological support should be offered to all women considering these options.

The management of ovarian cancer during pregnancy follows similar principles and depends on the tissue diagnosis; tumour differentiation; nodal status; tumour stage; and gestation of pregnancy. For patients with peritoneal spread or high‐risk early‐stage disease, neoadjuvant chemotherapy with pregnancy preservation may be feasible. A surgical approach may include both laparoscopic and open surgical techniques, with laparoscopic surgery associated with shorter operative time and reduced duration of stay [23].

Brain tumours during pregnancy are rare, occurring in approximately 4 in 100,000 pregnancies, and are associated with poor maternal and fetal outcomes [55]. There are no recommendations or consensus guidelines, and management will involve input from multiple specialties including neurology; neurosurgery; oncology; radiation oncology; palliative care; obstetrics; anaesthesia; and neonatology, on a case‐by‐case basis [56]. Physiological changes in the pregnant patient promote accelerated tumour growth with several theories proposed to explain this phenomenon: hormonal changes; increased levels of growth factors; angiogenic factors; and vascular endothelial growth factor have all been implicated [56]. Presenting symptoms such as headache, vomiting, dizziness and seizures may be difficult to differentiate from pregnancy‐related aetiologies creating a diagnostic challenge. Generally, any pregnant patient with focal, prolonged, progressive or unremitting neurologic symptoms should receive a full neurological evaluation as well as imaging for potential intracerebral pathology using CT or MRI modalities [56].

Treatment planning should prioritise selecting the best possible option for the patient as if they were not pregnant, with modifications aiming to reduce maternal and fetal risk. Systemic drug treatments may include corticosteroids to reduce cerebral oedema, with the added benefit of fetal lung maturation. Anti‐epileptic drugs may also be required, using the lowest effective dose; these should be supplemented with high‐dose folic acid (5 mg daily) in the first trimester, followed by low dose (0.4 mg daily) thereafter, due to their association with neural tube defects. Chemotherapy and radiotherapy may be used depending on tumour type.

Neurosurgical intervention in pregnancy should be considered for malignancies which are refractory to medical treatment or are life‐threatening due to factors such as impending herniation; mass effect; or intractable seizures [56]. Non‐emergent brain tumours should be resected in the second trimester as this carries the lowest risk of inducing preterm labour and intra‐operative haemorrhage. The general principles of neurosurgical and obstetric anaesthesia apply, including use of invasive arterial monitoring; maintenance of systolic blood pressure to baseline levels; maintenance of normocapnia; and left lateral tilt with the left lateral position preferred for intracranial procedures. If the fetus is viable at the time of planned neurosurgery, a decision must be made regarding the concurrent performance of caesarean birth. A risk–benefit assessment regarding the use of pharmacological thromboprophylaxis should be made in conjunction with the neurosurgical team.

Haematological malignancies in pregnancy are rare. Lymphomas (mainly Hodgkin's lymphoma) are the most frequently encountered cancers with an incidence of 1 in 6000 [57]. The therapeutic consensus for managing haematological malignancies during pregnancy prioritises the well‐being of the mother, while attempting to preserve the pregnancy and minimise fetal treatment‐related toxicity. Chemotherapy is the mainstay of treatment with an aim to administer the same treatment as would be given outside of pregnancy, wherever feasible. Anaesthetic implications relate mainly to the potential toxic effects of the chemotherapy regime, in particular neutropaenia and susceptibility to infection. If blood products are required, advice should be sought regarding the need for irradiated or cytomegalovirus‐negative products.

The management of metastatic disease in pregnancy depends on the underlying primary tumour type and extent of spread. In rare cases, placental and fetal metastases may occur and is most frequently seen with melanoma; leukaemias; lymphomas; and lung cancer. Such disease requires specialist management, including experts in maternal‐fetal medicine.

In conclusion, effective and safe anaesthetic management of cancer in pregnancy requires a multidisciplinary approach to balance maternal and fetal safety. Key management areas are summarised in Box 1. Advances in treatment options, including surgery and chemotherapy, have improved outcomes, though careful planning and individualised care are essential. Continued research and collaboration are needed to optimise protocols and support for affected women.

Box 1 **Summary of key considerations in the anaesthetic management of oncological disease in pregnancy**

**Management of pregnancy in patients with cancer**

■ Aim for birth at ≥ 37 weeks, unless clinical deterioration
■ Stop chemotherapy 3 weeks before birth
■ In most cases, vaginal birth is not contraindicated
■ Cell salvage is not contraindicated
■ In most cases, neuraxial techniques are safe and of benefit to patients
■ Caution with neuraxial techniques in vertebral/intracranial pathology or coagulopathy
■ In most cases, epidural blood patch is not contraindicated

**Management of cancer surgery in pregnant patients**

■ Do not delay investigation and staging of cancer in patients who are pregnant
■ Aim for surgery in the second trimester
■ Assess and optimise complications of chemotherapy and radiotherapy
■ If feasible, regional anaesthesia is preferred
■ For intra‐abdominal surgery consider risks of laparoscopic vs. open approach
■ Fetal monitoring before and after surgery according to gestational age should be led by the obstetric team
■ Prolonged venous thromboembolism prophylaxis may be required
